# Photonic Microfluidic Technologies for Phytoplankton Research

**DOI:** 10.3390/bios12111024

**Published:** 2022-11-16

**Authors:** José Francisco Algorri, Pablo Roldán-Varona, María Gabriela Fernández-Manteca, José Miguel López-Higuera, Luis Rodriguez-Cobo, Adolfo Cobo-García

**Affiliations:** 1 Photonics Engineering Group, Universidad de Cantabria, 39005 Santander, Spain; 2 CIBER de Bioingeniera, Biomateriales y Nanomedicina, Instituto de Salud Carlos III, 28029 Madrid, Spain; 3 Instituto de Investigación Sanitaria Valdecilla (IDIVAL), 39011 Santander, Spain

**Keywords:** phytoplankton, microfluidics, photonics

## Abstract

Phytoplankton is a crucial component for the correct functioning of different ecosystems, climate regulation and carbon reduction. Being at least a quarter of the biomass of the world’s vegetation, they produce approximately 50% of atmospheric O${}_{2}$ and remove nearly a third of the anthropogenic carbon released into the atmosphere through photosynthesis. In addition, they support directly or indirectly all the animals of the ocean and freshwater ecosystems, being the base of the food web. The importance of their measurement and identification has increased in the last years, becoming an essential consideration for marine management. The gold standard process used to identify and quantify phytoplankton is manual sample collection and microscopy-based identification, which is a tedious and time-consuming task and requires highly trained professionals. Microfluidic Lab-on-a-Chip technology represents a potential technical solution for environmental monitoring, for example, in situ quantifying toxic phytoplankton. Its main advantages are miniaturisation, portability, reduced reagent/sample consumption and cost reduction. In particular, photonic microfluidic chips that rely on optical sensing have emerged as powerful tools that can be used to identify and analyse phytoplankton with high specificity, sensitivity and throughput. In this review, we focus on recent advances in photonic microfluidic technologies for phytoplankton research. Different optical properties of phytoplankton, fabrication and sensing technologies will be reviewed. To conclude, current challenges and possible future directions will be discussed.

## 1. Introduction

Phytoplankton are all planktonic autotrophic aquatic organisms with photosynthetic capacity that live dispersed in the water. This name comes from the Greek terms, ϕυτoν (phyton, “plant”) and πλαγκτoς (“plánktos”, “wanderer” or “the one that wanders about”). They are also called microalgae, but despite all phytoplankton being microalgae, not all microalgae occur in plankton. Through photosynthesis, they produce energy-rich organic material that captures CO${}_{2}$ (almost a third of the anthropogenic carbon released into the atmosphere) and release oxygen (approximately 50% of atmospheric O${}_{2}$ [1]), thus helping to ameliorate greenhouse gases [2]. Phytoplankton is a taxonomically diverse group, consisting of more than ten thousand species and taxa that contribute to at least a quarter of the biomass of the world’s vegetation and constitute the base of the food web that supports either directly or indirectly all the animal populations of the open sea [3]. In addition, it has been introduced by some chefs into gourmet cooking recently [4]. As can be observed, phytoplankton is a key component for the correct functioning of different ecosystems, climate regulation and carbon reduction. The importance of its measurement and identification has increased in the last years, becoming an essential consideration for marine management [5]. Furthermore, due to climate change and human contamination [6,7] phytoplankton populations are being affected. Events in which phytoplankton undergo rapid population increase are known as algal blooms. A harmful algal bloom (HAB) occurs when it causes negative impacts by producing toxins, which can cause illness to mammals, fish, corals and other marine organisms [8,9,10]. These events are colloquially known as “red tides” since these organisms sometimes stain the water that colour. Therefore, HABs constitute a serious threat to public health, causing huge losses in fisheries and other productive sectors worldwide. The HAB problem is widespread on all the world’s seas and its trend is increasing. Red tides cause far-reaching economic damage to extractive and aquaculture activities. The group of phytoplankton that has caused the most toxic episodes is the group of dinoflagellates, specifically, Gymnodinus Gymnodinium catenatum and Gonyaulax tamarensis Lebour (both producers of paralytic toxin) as well as Dinophysis acuta Ehremberg and Dinophysis acuminata (responsible for gastrointestinal disorders due to shellfish ingestion). In many cases, the traditional classification of dinoflagellates based on morphological characteristics is insufficient. Therefore, it is necessary to develop other techniques to identify those harmful organisms. Another important application in which phytoplankton detection is gaining attention is in ships’ ballast water. Ballast water is used in ships to maintain safe operating conditions during transit. Although most phytoplankton die due to the environmental conditions in the ballast tank, some can survive. Due to these species that may survive and establish a reproductive population (with a huge number of phytoplankton and zooplankton), serious ecological, economic and health problems can be caused. This phenomenon was first studied between 1903 and 1907 in the North Sea [11]; in this article, Ostenfeld recognised the invasion of the Asian phytoplankton algae Odontella (Biddulphia Sinensis). Despite this, it was not until the 1970s that the scientific community reviewed the problem in detail. According to the International Maritime Organization (IMO), in the late 1980s, Canada and Australia were among the countries experiencing particular problems with invasive species. They brought their concerns to the attention of IMO’s Marine Environment Protection Committee (MEPC). Direct and indirect health effects are becoming increasingly serious and environmental damage is often irreversible. Recognising the possible severity of the consequences, action has been taken by the IMO, adopting the “International Convention on the Management of Ships Ballast Water and Sediments” [12]. Therefore, detecting microalgae and bacteria in the ship’s ballast water not only involves analysing the related quality of ballast water, but also is aimed at balancing the ecological environment and economic interests of each country [13].

As can be observed, the identification and measurement of specific characteristics of phytoplankton are essential for controlling pollution of the marine environment, as well as for aquaculture and the shellfish industry. The gold standard process used to identify and quantify phytoplankton is manual sample collection and microscopy-based identification, which is a tedious and time-consuming task and requires highly trained professionals. Flow cytometry is used to automatise the measuring process [14]. Flow cytometry gives the classification and identification of phytoplankton species, quantitative analysis and the extraction of parameters at the individual level. Generally, a typical flow cytometer can process thousands of cells per minute, much faster than manual observation using light microscopy. However, the expensive and bulky instruments make in situ measurement very difficult. For these reasons, the routine quantitative monitoring of phytoplankton in water is costly and challenging, requiring sophisticated equipment, a lab and almost unique expertise. Much recent activity has focused on developing microfabricated flow cytometers, which integrate inexpensive optical components to solve these problems. In fact, it can rapidly count cells and probe cellular populations at the single cell level [15]. Through microfabricated devices and microfluidic Lab-On-a-Chip (LOC) systems, it is possible to create a completely autonomous and portable integrated system. Microfluidic channels can handle tiny fluid volumes down to picoliters in a controlled microenvironment. For this reason, they allow the precise control and manipulation of fluids, typically in a passive way. Microfluidic LOC technology represents a potential technical solution for environmental monitoring, for example, identification and classification of particles in water [16,17] like phytoplankton. Its main advantages are miniaturisation, portability, reduced reagent/sample consumption and automation and cost reduction. Among all the microfluidic technologies [18,19,20,21], photonic microfluidic chips that rely on optical sensing have emerged as powerful tools that can be used to identify and analyse phytoplankton with high specificity, sensitivity and throughput.

Few literature reviews can be found related to microfluidics for marine research. For example, in [22], microfluidic systems for microalgal biotechnology are reviewed. Specifically, different applications are reported, e.g., microalgal biofuel applications, cultivation, downstream processing, microalgae-based microbial fuel cells and microalgae-based biosensors. A more general approach can be found in [23], focused on how microfluidic platforms address some challenges of plankton research. For example, analysis of a low density of organisms in environmental samples, difficulties in cultivating plankton, pre-concentrating, detecting and sorting them and how analytical microfluidic platforms are dedicated to the interactions between plankton and their environment. Finally, in a more recent work, the authors summarised some optofluidic systems and techniques for microalgal detection and characterisation [24]. For this reason, this review will focus only on recent developments in photonic microfluidics dedicated to phytoplankton research from a technological point of view. Optofluidic systems are the most widely used techniques for phytoplankton detection and characterisation. They are mainly based on three optical properties: fluorescence, scattering and imaging. Each of them can achieve cellular, lipid content, metabolic heterogeneity and count. This article is organised as follows: Section 2 will focus on the optical characteristics of phytoplankton (absorption, scattering and fluorescence) and Section 3 on the fabrication of microfluidic systems. Then, Section 4 will present the last advances in phytoplankton microfluidic technologies organised by phytoplankton optical properties. Finally, a discussion section will summarise and discuss the presented works. To conclude, we will present some current challenges and possible future directions of this promising technology.

## 2. Optical Characteristics of Phytoplankton

As mentioned before, the principal optical properties of phytoplankton are related to absorption, scattering and fluorescence. These optical processes are affected by the phytoplankton’s composition, specifically, many different pigments, with chlorophyll being the most important. Furthermore, depending on the group or taxon under consideration, the concentration of other pigments in their cells varies [25]. For this reason, phytoplankton has an important effect on the colour of the ocean and the measurement of these properties allows the study of their ecology and evolution over time.

### 2.1. Absorption

**Figure 1:**
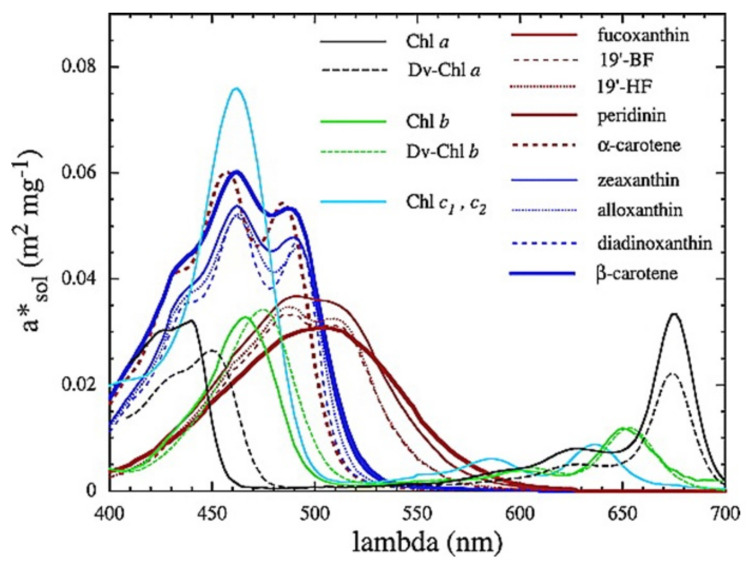
Weight-specific absorption spectra of the main pigments in vivo, $a_{sol,i}^{*}(\lambda$) (in m${}^{2}$ mg${}^{-1}$), which are derived from absorption spectra of individual pigments in solvent. The red and blue lines represent the absorption spectra of photosynthetic and nonphotosynthetic carotenoids, respectively. Reprinted with permission from Ref. [27]. Copyright 2004, American Geophysical Union.

Absorption is the process by which light is absorbed and converted into energy. This radiation, when absorbed, can be re-emitted or transformed into another type of energy, such as heat. Phytoplankton absorb sunlight and use this energy to produce chemical energy (photosynthesis). Two dominant peaks in the absorption spectrum exist in all phytoplankton cells (determined by chlorophylls). The primary one is in the blue (440 nm) and the second is in the red part of the spectrum (675 nm). Spectral light absorption curves in phytoplankton populations have been extensively studied (Babin et al. 2003), showing that the absorption coefficient ($a_{\phi }\left(\lambda \right)$) varies according to the presence of other pigments (depending on species and taxa) that will cause the blue peak to broaden and the appearance of additional absorption maxima. In addition, the packet effect due to phytoplankton size and changes in the physiological state of cells also affect these spectra [26,27]. These taxon-specific absorption peaks have been used as a tool for in situ optical detection [28], as well as for the development of remote sensing algorithms [29,30]. It has to be noted that absorption by phytoplankton is not a simple sum of the absorption coefficients of individual pigments. The absorption spectrum of phytoplankton varies in magnitude and shape due to the composition of different cell pigments [27]. In addition, specific proteins in the cells produce changes in the absorption spectrum, with cell pigment concentration and size also being influential [31]. However, as a simple approximation the so-called “package effect index”, $Q_{a}^{*}$(λ) (Equation (1)) is used.

(1) $$Q_{a}^{*}\left(\lambda \right)=\frac{a_{\phi }\left(\lambda \right)}{a_{sol}\left(\lambda \right)},$$

where $a_{sol}$(λ) is the absorption coefficient of the same material, which would be dispersed into the solution. The absorption coefficients $a_{sol}$(λ) (in m${}^{-1}$) can be obtained by summing the contributions of all individual pigments, using the relationship:

(2) $$a_{sol}\left(\lambda \right)=\sum C_{i}a_{sol,i}^{*}\left(\lambda \right),$$

where the $a_{sol,i}^{*}$ coefficients are the weight-specific absorption spectra of individual pigments (in m${}^{2}\cdot$mg${}^{-1}$) and $C_{i}$ are their concentrations in the medium (in mg·m${}^{-3}$). As stated in ref. [27], the $a_{sol,i}^{*}$ spectra of Figure 1 were estimated by scaling the absorption spectra of individual pigments in solvent, measured in relative values by high-pressure liquid chromatography, to the weight-specific absorption coefficients proposed in [32] and then shifting the positions of maxima to their in vivo positions, as in [33].

### 2.2. Light Scattering

**Figure 2:**
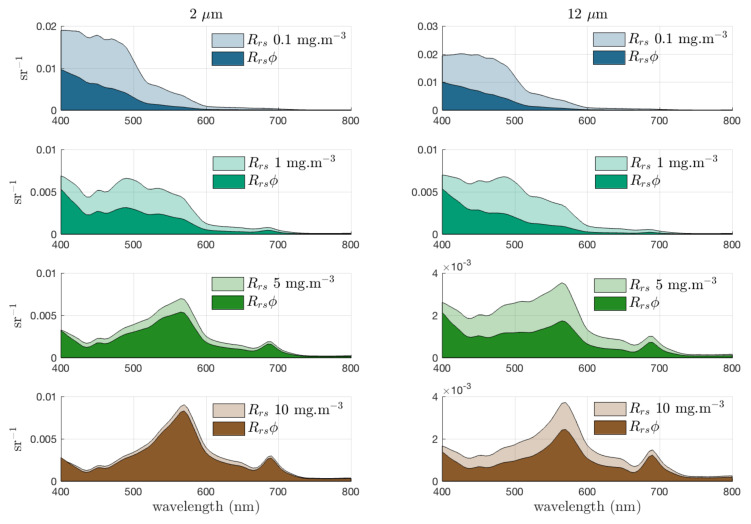
Relative contribution of phytoplankton to total $R_{rs}$ (with $a_{gd}\left(400\right)=0.07\cdot {\left[Clh_{a}\right]}^{0.75}$ and $b_{bnap}\left(550\right)=0.005$ m${}^{-1}$) for increasing biomass with effective diameter, $D_{eff}$ = 2 and 12 µm. The absorption caused by coloured dissolved organic matter ($a_{gd}$) covaries with $Chl_{a}$, whereas non-algal backscatter $b_{bnap}$ is constant. These populations are idealised examples and not intended to represent any observed relationship between $Chl_{a}$ concentration and $D_{eff}$. Reprinted with permission from [34]. Copyright 2018, MDPI.

When electromagnetic radiation strikes a molecule or particle, it can be scattered, i.e., the incident radiation is re-emitted in a direction different from that of the original radiation. In the case of elastic scattering (involving negligible energy transfer, same frequency), depending on the particle size with respect to the light wavelength (the colour), the re-emission intensity depends on the colour (Rayleigh scattering) or is independent of colour but has a certain direction (Mie scattering). Due to their morphology, phytoplankton cells are dominated by scattering or forward scattering rather than backscattering. Despite this, phytoplankton backscattering properties are also extremely important, as they are directly related to reflectance calculations, which is an essential measurement in oceanography [34]. In fact, the parameter “Remote Sensing Reflectance” ($R_{rs}$) is grossly proportional to backscattering divide by absorption ($b_{b}/a$) [35]. In Figure 2, the resulting contribution of phytoplankton $R_{rs}\left(\Phi \right)$ to the total $R_{rs}$ is shown. As can be observed, the contributions of the phytoplankton to the total, as a percentage of total $b_{b}/a$, vary with biomass (mg·m${}^{-3}$). Compared to the rest of the oceanic particles, the values of the scattering coefficients of phytoplankton are relatively low since they contain a large number of water molecules and exhibit strong absorption properties [36]. The exception to the rule is the coccolithophorid phytoplankton that produce small calcium carbonate flakes, which make them very effective dispersers and allow the blooms to be seen from space [37]. The phytoplankton scattering and backscattering coefficients and the scattering volume function can be obtained both from theoretical models (Mie theory) or measurements [38,39,40]. The properties depend on the size, shape and refractive index of all components of the phytoplankton cell [41]. Knowledge about the scattering angular distribution for phytoplankton is scarce due to the small number of experimental data sets [42,43]. Several commercial light scattering instruments for in situ measurements are reviewed by Moore et al. [44].

In recent years, Raman scattering has also been investigated as a possible technique in phytoplankton research. The Raman effect is the inelastic scattering of light (with different frequency) by a substance. The Raman shift is independent of the excitation wavelength, whereas the scattered light intensity is inversely proportional. The interest in the phenomenon lies in the fact that the colour (wavelength) change is characteristic of the substance and gives information about its composition, that is, the type of chemical bonds present and its atomic structure. For this reason, Raman spectroscopy was proposed as a fast and sensitive method to measure phytoplankton and its composition in marine environments [45]. The use of visible Raman spectroscopy to identify the nutrient composition or depletion, the detection of invasive, problematic phytoplankton species and cell viability have been reported in [45,46,47], respectively. However, there are some disadvantages when it is used to measure pigment-rich biological material. The most important is that Raman intensity is too low (only 1 in $10^{6}$–$10^{8}$ photons undergo Raman scattering) so the presence of fluorescence in phytoplankton (see next section) can mask the signals [48]. This effect can be reduced by using different excitation wavelengths and data processing (see Section 4.1). Another problem is that some pigment has a large range of bands while other biomolecule signals, such as fats and proteins can be masked [49]. Finally, the Raman spectra’s complexity limits chemometric methods’ application. Some recent works have proposed some solutions as using longer wavelengths (NIR) [50] and Fourier spectroscopy with multivariate data analysis [51] (see Section 4.2).

### 2.3. Fluorescence

Fluorescence is the property of some atoms and molecules to absorb light at a specific wavelength followed by light emission at a longer wavelength. The difference between scattering and fluorescence is their origin; whereas fluorescence occurs from a relatively long-lived excited electronic state, scattering occurs via the emission of a photon from a short-lived excited “virtual” state. In addition, the fluorescence emission wavelength is generally independent of the exciting wavelength, whereas light scattering increases with increases in the exciting wavelength. In the case of phytoplankton, several pigments (chlorophylls, pheopigments and phycobilins) fluoresce, with chlorophyll being the most important. One of the first proposals to use fluorescence as sensing parameter was presented in 1966 [52]. This work proposed the monitoring of phytoplankton biomasschlorophyll by fluorescence measurements. Nowadays, this technique is commonly used in commercial devices and sensors (fluorometers, radiance and irradiance meters, flow cytometers). One of the main advantages is the possibility of measuring from the sea surface by aeroplanes and satellites [53]. Although fluorescence spectroscopy can detect small concentrations of chlorophyll and other pigments, it is not very specific. The main problem is that phytoplankton cells’ fluorescence is highly variable due to their physiological conditions. Phytoplankton fluorescence depends on several parameters, e.g., the taxonomic position, the pigment content and ratio, the nutrient conditions, stage of growth, photoadaptation, physiological state, etc. [54,55,56]. Thus, deriving the biomass of spectral groups using the spectral fluorescence of multicomponent natural samples is not trivial [57]. For this reason, it is generally used to either approximate total plankton concentrations or detect the presence of harmful species; a recent comprehensive review can be found in ref. [56]. A simple equation can express fluorescence from phytoplankton chlorophyll, $F=I_{i}\cdot chl_{a}\cdot a_{phyto}^{*}\cdot \phi_{f}$ [58], with $I_{i}$ as the impinging light intensity, $chl_{a}$ as chlorophyll concentration, $a_{phyto}^{*}$ as chlorophyll-specific phytoplankton absorption coefficient and $\phi_{f}$ as the fluorescence quantum yield.

**Figure 3:**
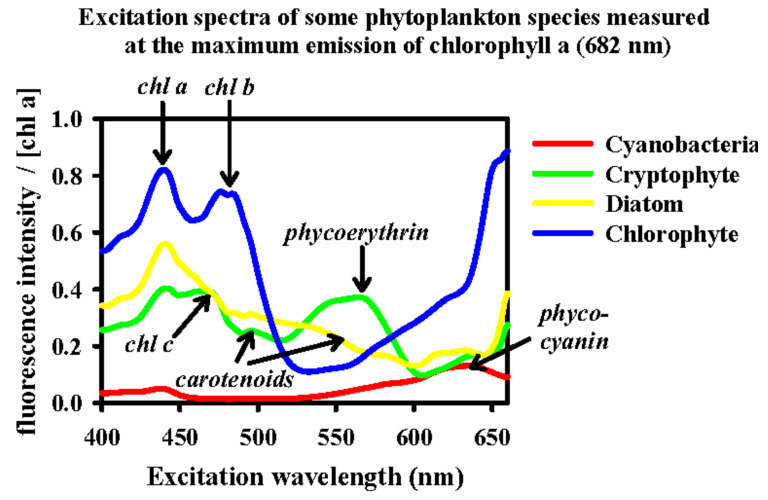
Fluorescence excitation spectra for different chemotaxonomic phytoplankton pigment groups. Reprinted with permission from [57]. Copyright 2003, International Council for the Exploration of the Sea (ICES).

As an example, in Figure 3, the spectral signature of some algal groups that mainly differ in their pigments can be observed. As mentioned in [57], all these measurements can be used as starting point to identify the phytoplankton.

## 3. Fabrication of Photonic Microfluidic Systems

A microfluidic system is based on a device in which one or more fluids flow through micrometer-sized channels ($10^{-4}$–$10^{-6}$ m). They can handle tiny fluid volumes down to picoliters in a controlled microenvironment; the flow is laminar and characterised by low thermal and chemical diffusion times. For this reason, they allow the precise control and manipulation of fluids, typically in a passive way, with some components being necessary such as micro-pumps or micro-valves for active devices [20]. Microfluidic chips or systems have applications in various fields, such as chemistry, environmental sciences or medical research. For example, microfluidic chips can be used to mimic the complex structure, microenvironment and physiological function of human organs [59], drug screening [60], toxicity testing [61] or stem cell testing [62], among other applications. In addition, microfludidic systems can be integrated with other photonic components to produce a photonic biosensor. Photonic biosensors have several advantages, such as immunity to electromagnetic interference, high-speed operation, low power consumption, use potential in harsh environments, miniaturisation capacity, multiplexing possibility, mechanical stability, low manufacturing and integration cost and real-time detection, directly and without labels (label-free) [63,64]. Photonic biosensors based on microfluidic channels are optical transducers. They modify a specific light parameter (intensity, phase or frequency) in the presence of an analyte. They typically consist of photodetectors (commonly used with light emission detection techniques, such as fluorescence), interferometers (with an analyte-sensitive arm) and/or resonant structures, such as microresonators (which also detect refractive index changes). All these sensors can be integrated on a micrometer scale, especially with the development of microfluidic systems in recent years.

The combination of microfluidics and photonics was proposed at the beginning of this century, when these two fields were maturing [65,66]. As mentioned below, despite the main fabrication techniques being based on lithographic methods, fs-lasers have been proven to fabricate microfluidic lasers [67], microfluidic channels and waveguides [68] in a 3D arrangement [69,70]. In addition, it is also possible to fabricate waveguides in commercial microfluidic chips by fs-laser, facilitating the fabrication process [71]. These advantages enable its use for LOC and bio-photonics applications [72]. Sensor characteristics are quantified in terms of selectivity (detecting a particular element) and sensitivity. Usually, to improve these parameters, receptors specific to the component to be measured are included. It should be noted that the comparison of the parameters reported in the literature is not simple, sometimes data are given in mass concentration (mass of a solute in relation to the volume of the solution) which is difficult to compare with other analytes or in the case of giving more comparable data, such as the refractive index units (RIU), it is not normalised to the size of the sensor. The most commonly used transduction signals in optical sensing are fluorescence, scattering and refractive index. There are two types of detectors, those specific to measuring the optical signals (fluorescence and scattering) or spectroscopic (label-free, to measure refractive index changes). In the first case, fluorescence has the highest sensitivity, down to the single-cell limit. Scattering, however, can be masked from the previous signal (see more details in the following sections). These systems can be supported by engineered microfluidic channels that stretch and sort the phytoplankton cells [23,73]. In the case of LOC interferometric sensors, a miniaturised system is used, with two separate arms in which one is the reference and the other is the measurement arm (in which the receiver and the analyte are included). When the analyte is on the reference arm, the light passing through it will have a phase difference with respect to the light from the other arm. When the light from the two arms recombines, it can be measured to measure the phase delay, which will be greater the higher the rate of the analyte. Although it is difficult to achieve the same selectivity and sensitivity as the previous sensors, they are widely used in environmental measurement applications such as air quality, greenhouse gases, detection of chemical, biological, radiological, nuclear, explosive agents, etc. [74]. In the case of phytoplankton, no sensors have been reported yet.

**Figure 4:**
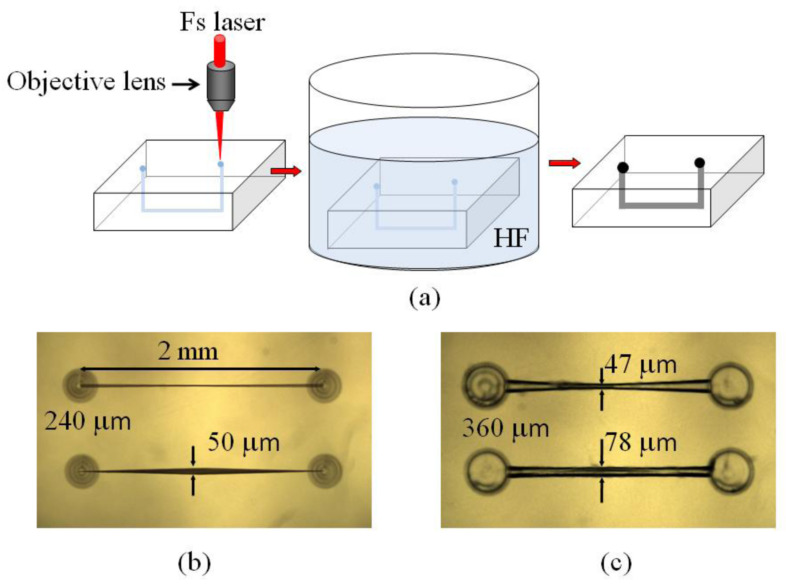
Femtosecond laser microfabrication of opto-microfluidic devices: (**a**) fabrication procedures, (**b**) U-shape-microchannels in fused silica before etching, and (**c**) after etching for 5 h in 20% HF acid within a shaker. Reprinted with permission from [75]. Copyright 2011, MDPI.

**Figure 5:**
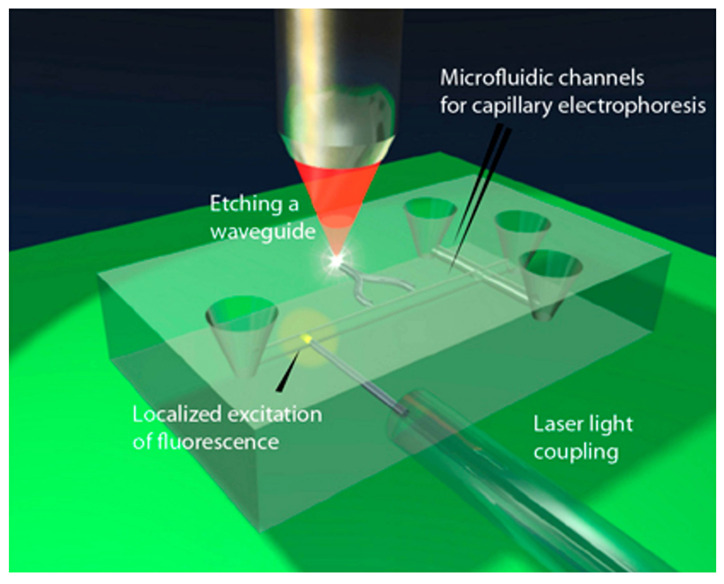
A schematic representation of an LOC device designed for capillary electrophoresis fabricated with a femtosecond-laser. The fluorescence is excited in a highly localised region in the microchannel by the optical waveguides and optical fibres. Moreover, the waveguides guide emissions to a detector. Reprinted with permission from [76]. Copyright 2014, SPIE.

The fabrication processes in glass or silicon depend on the type of fabricated structure; extended details can be found in [20,76]. For example, soft lithography techniques have been mainly used to make microfluidic channels [77]. For this, polydimethylsiloxane (PDMS) elastomer can be used to have a fast and cheap fabrication process. However, the main problem is fabricating 3D structures, which requires additional stacking and bonding processes [20]. The same problem can be found in other typical techniques such as planar microfabrication (e.g., injection moulding or semiconductor processes based on photolithography). To solve this problem, femtosecond (fs) laser fabrication is the best option, as has been demonstrated in the 3D fabrication of transparent structures [78,79]. Some of the most important characteristics of fs-laser are: it is a single-step and maskless process, it can be applied to several materials (e.g., glasses, crystals, polymers) by changing the irradiation parameters; it can be used in 3D as depth irradiation can be easily modified; the fabrication of several components in different steps is possible. As an example, a schematic depiction of the fabrication procedure can be found in Figure 4a. Figure 4b,c show real examples of fabricated devices and Figure 5 illustrates how optical waveguides are integrated into a commercial LOC.

In the case of microfluidic channels and other optical components (e.g., microlenses, hollow optical waveguides, optical micro-resonators [80,81,82]), the fabrication process involves the use of a wet chemical etching (HF or KOH) after the application of fs-laser [83]. KOH can produce defects such as relatively high surface roughness (of a few tens of nanometers). Despite this, this can be solved by polishing the accessible regions or using post-processing heat techniques for internal walls, e.g., oxygen/hydrogen flame polishing [80], annealing in an oven [82] and CO${}_{2}$ laser reflow [84]. Nowadays, the main accepted hypothesis for the etching rate variation in fused-silica regions where the fs-laser is applied is that the laser beam reduces the Si-O-Si bond angle [85]. Other studies have tested the effect of the polarisation in the etching selectivity, as the absorption of the laser energy is spatially modulated, producing the so-called nanogratings perpendicular to the polarisation direction that enhances the etching selectivity [86]. Another option to avoid etching is fs-laser drilling of glass immersed in distilled water (liquid-assisted fs-laser drilling) [87]. In this case, the immersion is intended to remove some of the ablation debris that can contaminate the channel and restrict the size of the microstructures [88]. Some works have also proposed using porous glass (10 nm pores uniformly distributed) [89]. The process avoids wet etching and the porous element can be sealed by annealing the glass. Compared to fused silica, the channels can have arbitrary geometries, unlimited lengths and features sized beyond the optical diffraction limit [20]. Finally, it has to be noted that fs-laser can also produce typical waveguides based on refractive index modulation, which is caused by a localised nonlinear absorption at the laser focus region. Low index contrast waveguides have both a low transmission loss and excellent mode overlap with optical fibre, meaning that the overall extraction efficiency of a circuit is high [90]. When low intensities are used, the temperature rises and decreases with each pulse, resulting in a refractive index change that depends on the glass density and cooling rate. For higher intensities, the mechanism is based on the plasma formed inside the glass, which creates a Coulomb explosion and a shockwave; this induces an inhomogeneous material distribution and a refractive index change. Several works have demonstrated the feasibility of an fs-laser in terms of creating waveguides in different glasses [90,91,92,93,94]. For example, in [91] waveguides in fused silica glass are demonstrated with an RI change of $∼4\times 10^{-3}$) and low propagation loss of 0.12 dB/cm. However, some issues such as spherical aberration, self-focusing and nonlinear absorption are common and produce an asymmetrical mode field pattern [94,95,96]. To control this parameter, the use of spatio temporally focused beams [97], spatial light modulators [98,99], thermal annealing [100] or polarisation control [101] have been proposed. As can be observed, the field has been very active this last decade.

## 4. Phytoplankton Microfluidic Technologies

As mentioned before, the most traditional method for identifying and quantifying phytoplankton is manual sample collection and microscopy-based identification, which is a tedious and time-consuming task and requires highly trained professionals. Standard flow cytometry has recently been used to automatise the phytoplankton measuring process. However, the expensive and bulky instruments make the in situ measurement very difficult. Microfluidic flow cytometry provides a solution by producing portable devices that enable on-site phytoplankton analysis and classification. However, conventional microfluidic flow cytometry generally relies on light scattering (Section 4.1) or fluorescence properties (Section 4.2), which cannot offer spatially resolved characterisation and distinguish through differences in surface morphology. For these reasons, imaging-based flow cytometry has been recently proposed to enhance phytoplankton measurement (Section 4.3). Impedance measurements sometimes support these techniques, so a brief review is also included at the end of this section (Section 4.4).

### 4.1. Technologies Based on Scattering

Absorption, scattering and side scattering of phytoplankton have been traditionally used in their characterisation (see Section 2). The cell properties are also measured in commercial flow cytometers using scattered light. Being an elastic scattering process, the measured wavelength will be the same as the source. The forward and side light scattering is collected from narrow angles and light diffracted around the cell, respectively. These measurements are demonstrated to give information about cell size and shape. Despite this, the measurement of these properties in LOC devices is not so usual and they are used in combination with other techniques.

**Figure 6:**
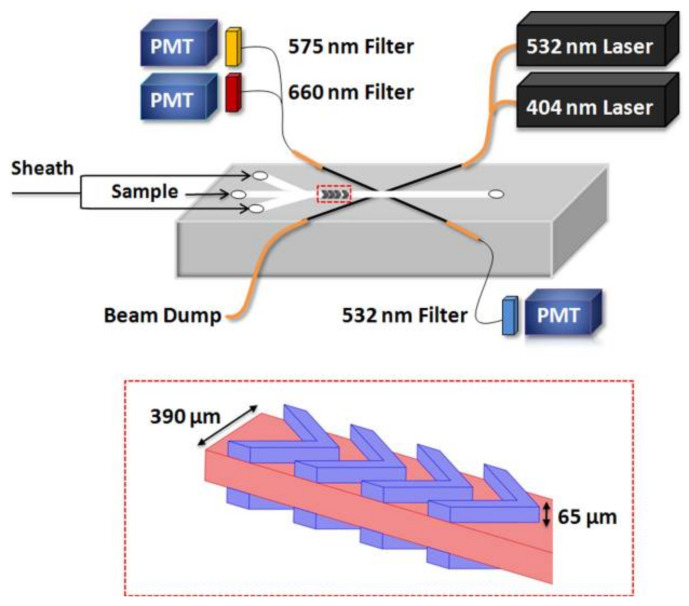
A schematic depiction of the proposed cytometer. (Top) The optical and microfluidics setup. (Bottom) A zoomed-in view showing the chevron grooves extending into the PDMS substrate. Reprinted with permission from [102]. Copyright 2011, AIP Publishing.

For example, in [103], Hashemi et al. developed a lab on chip based on autofluorescence and light scattering, which consisted of a special microfluidic chip with chevron grooves that can focus the flow in two dimensions. By using two grooves (on the top and bottom of the microchannel), they created two symmetrical sheath flows that wrap around a central flow (see Figure 6 bottom). A 488 nm argon laser and three photomultiplier tubes were used to record the fluorescence and light scattering signals. The proposed system was capable of detecting the picoplankton *Synechococcus* sp.with diameters lower than 1 µm and phytoplankton species as long as 80 µm (Nitzschia d.). In a later work, the authors replaced the large 488 nm argon laser with two solid-state laser sources (404 nm and one 532 nm), which are both small for in situ measurement (see Figure 6 top). In addition, the provided wavelength is closer to the maximum absorbance of chlorophyll and phycoerythrin [102].

**Figure 7:**
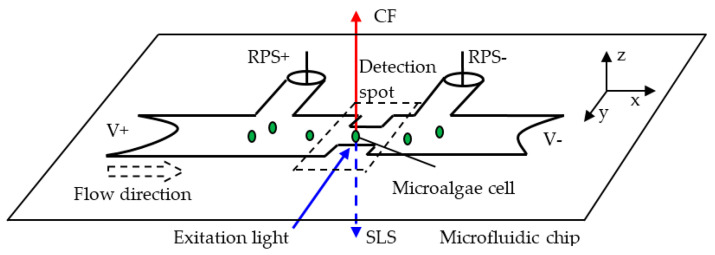
Schematic representation of the operation principle of single microalgae cell classification. The system is based on the simultaneous detection of chlorophyll fluorescence (CF), side light scattering (SLS) and resistance pulse sensing (RPS) signals. Reprinted with permission from [104]. Copyright 2016, MDPI.

In ref. [104], side light scattering is measured in combination with chlorophyll fluorescence and resistance pulse sensing (RPS) (see following sections). As shown in Figure 7, the chlorophyll fluorescence with 680 nm wavelength is detected from the positive Z-axis and the side light scattering with 480 nm wavelength is detected from the negative Z-axis. At the same time, the signal of resistance pulse sensing of the phytoplankton is acquired from the difference between the two sense arms of RPS+ and RPS− [104]. The light scattering measurements provide information about the intracellular contents and the size and surface roughness of the cell. On the other hand, the chlorophyll fluorescence is related to the activity and the RPS to the size of the cell.

**Figure 8:**
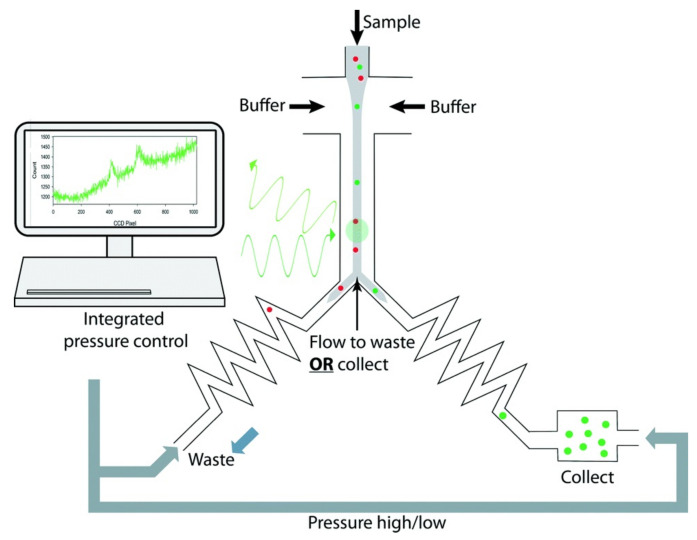
Schematic representation of the proposed device. A stream of cells were hydrodynamically focused in the detection channel for continuous Raman acquisition; on-the-fly classification was carried out to identify target cells and was immediately followed by alternating the pressures applied to the waste and collection channels, to direct the target cells to the collection chamber. Integrated software was developed to synchronise and automate all the operations. Reprinted with permission from [111]. Copyright 2016, Royal Society of Chemistry.

As mentioned in Section 2.2, in recent years, Raman scattering has also been proposed as a possible technique for phytoplankton research [45,105,106]. In the case of microfluidic devices, Raman has been proposed in several works to act as a sorting method (Raman-activated cell sorting, RACS) [107]. Despite this, Raman signals are too low, requiring long interrogation times as compared with fluorescence detection (in the order of seconds to minutes in comparison with microseconds in fluorescence). One solution can be the isolation [108] or immobilisation [109] of single cells. In the first work, a highly motile species (Euglena Gracilis) is isolated by semiclosed microchannels with liquid flow only, whereas in the second one, optical tweezers are used (Raman tweezers, see [110] for more information). Another innovative solution was a “trap-free” RACS in a flow that allows continuous and automated sorting of individual cells [111]. In this case, the authors provide a stable flow field in the detection region by using two pressure dividers that eliminate local pressure fluctuations (see Figure 8). They achieved a 96.3% purity of the selected cells at a speed of 0.5 Hz.

Another proposed technique to improve the throughput has been Raman-activated droplet sorting (RADS). For example, Kim et al. recently reported a method that uses Raman spectroscopy with PDMS-based microfluidic devices to perform on-chip, droplet-based in vivo phytoplankton lipid analysis with single-cell resolution [112]. The time-course tracking and study of lipid accumulation in C. reinhardtii cells under eight different culture conditions was successfully conducted, demonstrating the potential for Raman-microfluidics-based lipidomics. Another RADS microfluidic system for functional screening of live cells in a label-free and high-throughput manner was presented in [113]. In this case, the sorting is achieved by dielectrophoresis. The system is based on the level of astaxanthin content within phytoplankton cells, which has a high detection and sorting efficiency of approximately 260 cells/min and high accuracy of 98%. Furthermore, 92.73% of the selected cells remained alive and could proliferate. Haematococcus pluvialis cells in the microchannel were hydrodynamically focused in a single line after squeezing two buffer streams. When the cell passed through the detection region, the astaxanthin content in the cell was measured with Raman spectroscopy; then, the detected cell was encapsulated in the droplet for sorting the next step. Positive dielectrophoresis was used to manipulate the cell in the droplet with efficient trap and release, thus forcing cells with different astaxanthin contents into the pre-designed collection channel or waste channel based on their Raman spectroscopic responses.

### 4.2. Technologies Based on Fluorescence

Optical fluorescence-based detection methods are among the most popular and widely used in the detection and characterisation of biological and biochemical samples in microfluidic chips, including phytoplankton analysis. The fluorescence of phytoplankton can be the result of endogenous pigments (see Section 2.2) and different species of phytoplankton have their unique fluorescence spectra due to the different pigment ratios. The most common technique that uses fluorescence properties is flow cytometry. A comprehensive review can be found in [14]. However, the introduction of microfluidics reduces the device size through LOC devices. Based on this principle, Benazzi et al. developed a high-speed microfluidic platform to measure fluorescence from single cells at three different wavelength ranges (using a luminescence spectrometer with a 532 and 633 nm laser), achieving cell discrimination at a flow rate of 3 cm·s${}^{-1}$ [114].

**Figure 9:**
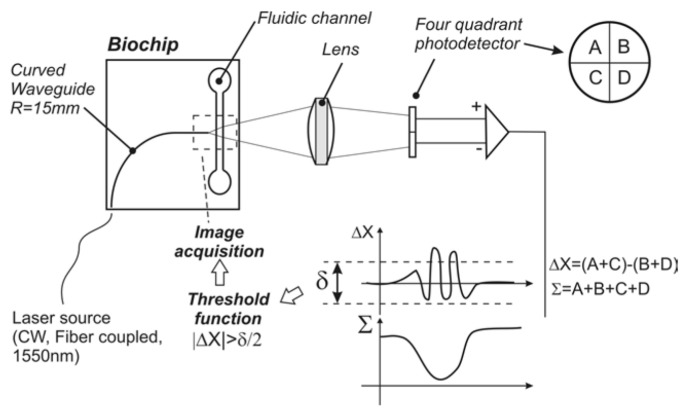
Schematic of biochip working principle: the biochip consists of a fluidic channel and a curved waveguide buried in the glass. A Gaussian beam emitted by a single-mode fibre is coupled into the biochip waveguide and diverges to illuminate a small length of the fluidic channel. Objects that pass through the fluidic channel momentarily distort the beam intensity profile. The light from the biochips is then refocused onto a four-quad detector to monitor small intensity changes. Reprinted with permission from [115]. Copyright 2011, Optica Publishing Group.

Some years later, Schaap et al. [115] developed an LOC that could distinguish phytoplankton species with a more straightforward setup. Only one focalised laser source and a single quadrant-cell photodetector were utilised (see Figure 9). A curved waveguide guided the laser light to the microchannel and the different cells produced distinctive wavelets dependent on the phytoplankton geometry and size. As mentioned in the previous section, the same year Hashemi et al. developed another autofluorescence-based lab on chip which consisted of an optofluidic cytometer [102,103]. As stated by the authors, the differences in fluorescence signals were used to reveal the different ratios of chlorophyll and phycobilins. In contrast, the differences in light scattering signals were used to assess the size and shape of phytoplankton cells. Even the smallest species, with a size of 1 µm could be identified (*Synechococcus* sp.). A few years later, Wang et al. [116] proposed a simple optofluidic device for fluorescence-based phytoplankton detection. It consisted of one sample channel between two sheath channels. Thanks to this configuration, the two branch channels’ laminar flows forced the main channel’s phytoplankton cells to line up into one line. A 488 nm laser diode was used as the excitation light to illuminate the sample cells. A photodiode was selected to measure the fluorescence of chlorophyll with an output voltage corresponding to the intensity of the fluorescence. It can be used to identify dead cells and living cells by calibrating the fluorescence intensity. The results confirmed that the developed system based on chlorophyll fluorescence could not only detect the living status of single phytoplankton cells but also can evaluate their viability quantitatively [116]. The same principle was subsequently used in combination with impedance measurements [117]. In recent work, the authors developed a ballast water rapid detection device based on the previous fluorescence microfluidic sensor [118]. The authors concluded that obtained results agree with the laboratory standard test measurements, with the advantage of on-site real-time ballast water detection. Another interesting study was presented in [119]. In that work, a label-free analysis and sorting of phytoplankton in microdroplets by chlorophyll fluorescence was presented.

**Figure 10:**
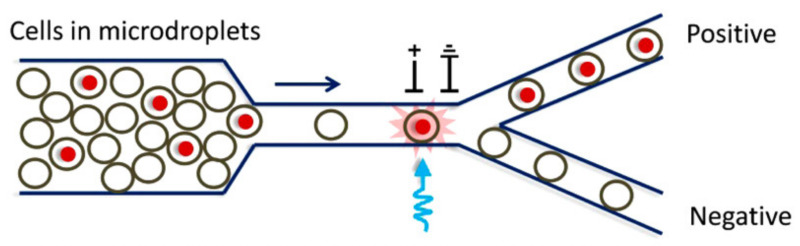
Schematic depiction of the fluorescence-based sorting method. Reprinted with permission from [119]. Copyright 2016, ACS.

As can be observed in Figure 10, the electrical signal of the detector was used to trigger a deflecting system based on voltage. As the authors state, this technique can be applied as a screening tool for microalgal libraries. Moreover, as the method allows the measurement of intrinsic chlorophyll per cell and total chlorophyll per droplet, the cell number and biomass evolution over time can be measured.

### 4.3. Imaging Flow Cytometry Technologies

As mentioned before, a conventional cytometer generally relies on phytoplankton’s light scattering or fluorescence properties, which cannot offer spatial size characterisation and distinguish through differences in surface morphologies. To overcome this challenge, imaging flow cytometry (IFC) was first proposed in 1979 [120] and further developed in the 1980s [121]. Thanks to microfluidics, image-based microfluidic flow cytometers have been recently proposed to create novel and high-efficiency platforms that combine the high-throughput nature of conventional flow cytometry techniques with the optical resolution of microscopy [15].

This technique combines speed and significant sample size capabilities of flow cytometry and zooming capabilities of microscopy (up to 60×), contributing significantly to the advancement of phytoplankton analysis [122]. Using this technique, the measurement of phytoplankton morphology, cellular processes, cell-to-cell interactions, population dynamics and ecology has been improved [123]. Still, there are some issues to be solved, for example, their low analytical throughput (typically between 2000 and 3000 cells/s at 20× magnification); more than one order of magnitude lower than non-imaging flow cytometers [124]. Moreover, the limited depth of field of the objective causes a limitation in the size of the channel and measured cells, requiring other methods such as acoustic focusing to improve it [125]. Finally, the bulky devices make it difficult to use them in situ. To solve the issues mentioned above, different solutions have been proposed [124]. Despite this, for phytoplankton there are additional issues such as the considerable amount of different species in the same sample. In [122,123], other applications of IFC technology for analysing microalgae cultures and phytoplankton are thoroughly reviewed (until 2017). Ref. [126] systematically reviews articles from 2017 to 2020 using the commercial device FlowCam for phytoplankton research. For this reason, in this review, we will focus only on novel proposals of the last five years.

**Figure 11:**
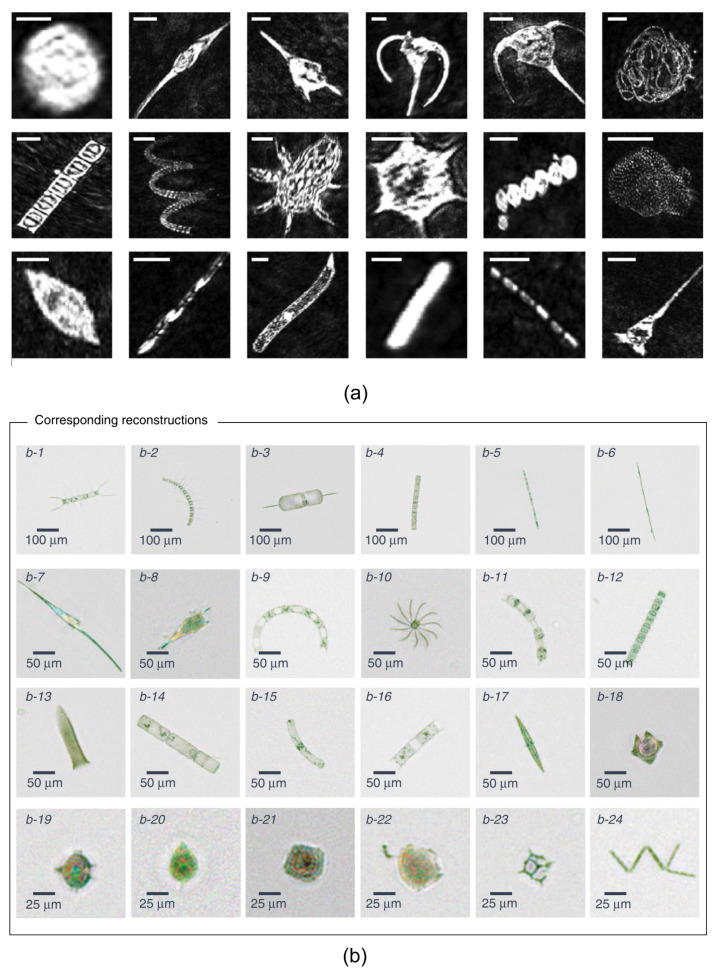
Examples of various ocean planktons. (**a**) Amplitude images reconstructed and detected from specific focal planes for each plankton class. From top left to lower right: *Alexandrium tamarense, Ceratium fusus, Ceratium lineatum, Ceratium longpipes, Ceratium *sp., *Chaetoceros socialis, Chaetoceros straight, Chaetoceros *sp., *Crustacean, Dictyocha speculum, Melosira octagona, Parvicorbicula socialis, Prorocentrum micans, Pseudo-nitchzia arctica, Rhizosolenia setigera, Rods, Skeletonema costatum, Tintinnid.* All images are segmented to 128 × 128 pixels and scale bars represent 50 µm. (**b**) Planktons detected at the Los Angeles coastline, represented by their phase-contrast reconstructions following phase recovery. The organisms were identified as (**b1**) Chaetoceros lorenzianus, (**b2**) Chaetoceros debilis, (**b3**) Ditylum brightwellii, (**b4**) Lauderia, (**b5**) Leptocylindrus, (**b6**) Pseudo-nitzschia, (**b7**) Ceratium fusus, (**b8**) Ceratium furca, (**b9**) Eucampia cornuta, (**b10**) Bacteriastrum, (**b11**) Hemiaulus, (**b12**) Skeletonema, (**b13**) Ciliate, (**b14**) Cerataulina, (**b15**) Guinardia striata, (**b16**) Lithodesmium, (**b17**) Pleurosigma, (**b18**) Protoperidinium claudicans, (**b19**) Protoperidinium steinii, (**b20**) Prorocentrum micans, (**b21**) Lingulodinium polyedra, (**b22**) Dinophysis, (**b23**) Dictyocha fibula (silica skeleton) and (**b24**) Thalassionema. Reprinted from (**a**) [129] Copyright 2021 (**b**) [127], Copyright 2018, Springer Nature.

Holographic and multispectral techniques have been proposed to implement different devices. In 2018, a holographic device capable of detecting phytoplankton flowing through a 0.8 mm thick microfluidic chip was proposed (Figure 11b) [127]. A deep convolutive network is used to reconstruct the acquired holograms automatically. This device allows the real-time imaging of highly dense samples. Specifically, 24 microalgae species were identified in flow-through water samples with a high flow rate of 100 mL/h. In addition, the concentration measurement of the potentially harmful microalgae Pseudo-nitzschia is also reported. In 2021, the same authors proposed new analysis methods to perform an automated and high-throughput phenotypic inspection of microalgae populations in the presence of pollutants within the water sample [128].

The same year, using holography and deep learning, the capability to produce and reconstruct sharp images of important plankton groups from both culture and environmental samples was demonstrated [129] (Figure 11a). On the topic of imaging flow cytometry combined with deep learning, the following recent papers demonstrated the efficiency of those techniques for identification and classification of protozoa [130,131]. They have the potential of being extended to the identification of classification of phytoplankton.

One problem of previous systems is that current optical imaging technologies still lack the practical speed and sensitivity for measuring thousands to millions of cells down to single-cell precision [132]. A solution to this problem is optofluidic time-stretch microscopy, which can perform high-throughput imaging flow cytometry up to 100,000 cells per second. Since it was first demonstrated in 2009 [133], this technique has been demonstrated in on-chip microfluidics for several applications. In the case of phytoplankton, several works have been reported since 2016 [134,135,136,137,138,139,140]. This kind of system is composed of several key components, i.e., a broadband pulsed laser (fs-laser), a temporal disperser (usually a long disperser fibre optic), two spatial dispersers (diffraction gratings), two objective lenses, a microfluidic device, a single-pixel photodetector, an oscilloscope and a digital signal processor. For this reason, LOC systems can be limited by the size of some of the components mentioned above. Readers can check ref. [141] for more information and detailed instructions to fabricate an optofluidic time-stretch microscopy and measure cells in microfluidic channels.

### 4.4. Electrochemical Impedance Spectroscopy

**Figure 12:**
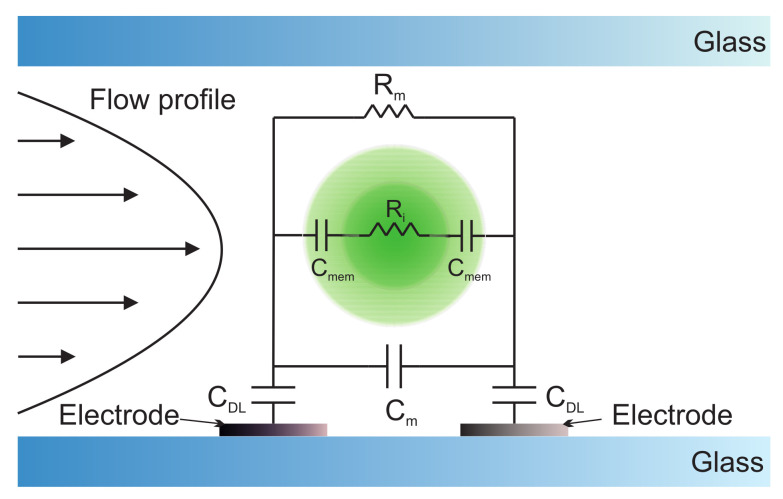
Equivalent electrical circuit of a phytoplankton cell in a microfluidic channel. $R_{m}$ and $C_{m}$ are the resistance and capacitance of the medium, respectively, $C_{mem}$ is the capacitance of the cell membrane, $R_{i}$ is the resistance of the cell cytoplasm and $C_{DL}$ the capacitance of the electrical double layer (DL). The values of the individual electrical components are determined by the dielectric properties of the suspending medium, the geometry of the chip and the dielectric properties of an individual cell [114].

Although the measurement of dielectric properties of phytoplankton does not fall within the scope of this review, we have briefly included it because recently proposed devices employ multiparametric sensing, combining dielectric and optical properties. One of the most used techniques to characterise the electric properties of electrochemical systems is electrochemical impedance spectroscopy (EIS). It can characterise the dynamics of bound or mobile charges in the bulk or interfacial regions of any solid or liquid material (ionic, semiconductor and dielectrics). The main parameter EIS gives impedance, a concept proposed by Oliver Heaviside in 1880. Impedance is the complex ratio of the voltage to the current for an alternating current, Z=V(ω)/I(ω), translating the concept of resistance to AC systems (possessing both magnitude and phase). In recent years, this technique has grown tremendously and is now widely employed in various scientific fields. The medical field is one of the most demanding sectors for this technique: clinical scales to measure corporal parameters, the efficacy of medicinal products [142], cancer detection [143], etc. Another important sector is material engineering: the study of new materials, batteries, metals (corrosion process) [144], etc. In addition, there is a growing interest in using this technique in bioengineering [145]. This technique gives useful information about tissue or cells. The operation principle of single-cell impedance spectroscopy for high-speed analysis has been reported since the beginning of the century [146,147,148]. Some comprehensive reviews can be found in [149,150]. In the simplest case, phytoplankton behaves like a spherical shell, describing each cell as a perfect sphere with a conductive outer shell and membrane and a resistive interior (see Figure 12) [114]. For other types of phytoplankton, this model has to be modified, e.g., phytoplankton without cell walls or with other biological configurations.

As the frequency response of phytoplankton is complex, the EIS technique is appropriate for characterising their impedance. For example, in [114], different phytoplanktonic species’ impedances were measured (*Isochrysis galbana, Synechococcus* sp. and *Rhodosorus m*.). The authors conclude that low-frequency signals could be used to measure the size of the particles, but the system is not sufficiently sensitive to detect the smaller cells (<2 µm). In more recent work, phytoplankton bioimpedance was performed using interdigitated electrodes and an impedance analyser [151]. The results showed no significant difference in the extracted cytoplasm conductivity, whereas the specific membrane capacitance (membrane capacitance per unit area) between Chlamydomonas and Selenastrum cells differed significantly.

Another technique that simplifies the system (an impedance analyser is unnecessary) is Resistive Pulse Sensing, which is based on the Coulter principle. Particles in low concentrations suspended in the medium can be counted by passing them through a microfluidic channel. A particle passing through the channel displaces a volume of medium equivalent to the submerged volume of the particle from the detection zone. This causes a short-term change in impedance across the aperture. This change can be measured as a voltage or current pulse. The height of the pulse is proportional to the volume of the detected particle. Assuming a constant particle density, the pulse height is also proportional to the particle’s mass. For example, this technique was demonstrated by Song et al. [152,153] in an analysis of ships’ ballast water, detecting and counting phytoplankton cells and measuring their sizes. In a subsequent work [117], the combination with chlorophyll autofluorescence intensity detection allows for measuring cell viability, excluding interference of other particles and dead cells and producing more accurate results. The system was integrated into an underwater device with advantages such as automation, portability, low cost and easy operation. After that, the system continued being tested to comply with the Standard D-2 performance [13].

One drawback of this kind of system is the lack of close contact between cells and electrodes when passing through the microchannel. This issue could lead to current leakage where electric signals circumvent the cells under measurement by travelling through solutions surrounding the cells [150]. Another issue could be the position dependence of the measurements and the overlapping of two or more cells in the channel. One solution could be the use of narrower channels and sorting methods. In this regard, sorting plankton in microfluidic devices is an effervescent research field where numerous methods have been developed and are continuously improved through the years and applications [23]. The topic is thoroughly reviewed in the latter reference.

## 5. Discussion

The identification and measurement of specific characteristics of phytoplankton are essential for controlling pollution of the marine environment and aquaculture and the shellfish industry. Although manual sample collection and microscopy-based identification is the traditional method to measure phytoplankton, some flow cytometry techniques have improved the throughput. However, the expensive and bulky instruments make in situ measurement difficult. Precise control, manipulation and measurement are possible through LOC systems incorporating microlfuidic channels and basic optical components. Several techniques are used in this kind of device, along with their advantages and drawbacks. Moreover, each can detect different parameters of the phytoplankton, so their combination is used in several proposed devices (see Table 1).

For example, scattering-based technologies can measure the size and surface roughness of the cell; they are used in remote measurements but also in microfluidic channels. The forward and side light scattering is collected from narrow angles and light diffracted around the cell, respectively. These measurements are demonstrated to give information about cell size and shape. Despite this, the low intensity of the signal with respect to fluorescence can be a drawback. For this reason, the measurement of these properties in LOC devices is not so usual and when they are used, they are in combination with other techniques. On the other hand, microfluidic technology combined with Raman spectroscopy precisely identifies the phytoplankton composition and nutrient contents. However, the low intensity of the Raman signal in comparison with normal scattering and fluorescence (only 1 in $10^{6}$–$10^{8}$ photons undergoes Raman scattering) leads to errors during data analysis, limiting the measure to species with low fluorescence. This effect can be reduced by using different excitation wavelengths and data processing. Another problem is that some pigments have an extensive range of bands while other biomolecule signals, such as fats and proteins, can be masked. Another consideration is that PDMS is not recommended to fabricate the microfluidic channels as this material can also generate Raman scattering. Finally, the Raman spectra’s complexity limits the application of chemometric methods. As mentioned before, recent works have proposed some solutions using longer wavelengths (NIR) and Fourier spectroscopy with multivariate data analysis. To increase the throughput, some solutions have been the isolation or immobilisation of single cells and droplet microfluidic devices (RADS).

In the case of fluorescence, phytoplankton have their unique fluorescence spectra due to the different pigment ratios. For this reason, it can be combined with other techniques to classify species. Fluorescence spectroscopy can detect small concentrations of chlorophyll and other pigments, but it is not very specific. The main problem is that phytoplankton cells’ fluorescence is highly variable due to their physiological conditions. Phytoplankton fluorescence depends on several parameters, e.g., the taxonomic position, the pigment content and ratio, the nutrient conditions, the growth stage, photoadaptation and physiological state. Thus, deriving the biomass of spectral groups using the spectral fluorescence of multicomponent natural samples is not trivial. In the case of microfluidics, the size reduction increases the throughput; some works have demonstrated the detection of the living status of single phytoplankton cells and their viability quantitatively. In addition, due to the sort interrogation times, it can be used in sorting devices.

By leveraging algorithms such as deep learning and compression detection, flow cytometry based on microfluidic imaging could perform the powerful function of automatically identifying and counting phytoplankton. Particularly enabled by new multifunctional imaging techniques, the microfluidic flow cytometer can offer information regarding cell morphology, intracellular lipid and pigment. Compared to microfluidic devices based on fluorescence detection and Raman spectroscopy, the imaging-based flow cytometer offers flexibility and feasibility to perform machine automation without human intervention. On the other hand, the automatic identification and classification of the phytoplankton require a database for machine training. Therefore the demand for sample preparatory images based on high experience must be implemented before on-site use. Another issue is the limitation of processed cells per second due to the limited capture speed of current CCD and CMOS systems (limited throughput). A solution to this problem can be optofluidic time-stretch microscopy, which can perform high-throughput imaging flow cytometry up to 100,000 cells per second. However, the complexity of the setup (fs-laser, dispersers, etc.) makes it difficult to make portable devices.

Finally, impedance measurements can be an excellent complementary option, as they can be combined perfectly with previous techniques. The measurements can give information about cytoplasm conductivity and membrane capacitance, producing a characteristic signature for each species. The main problem is that phytoplankton without cell walls or with other biologic configurations are challenging to model. Another inconvenience is the requirement of impedance analysers that can be bulky for portable systems; despite this, custom circuits could be made to solve this issue. On the other hand, to simplify the setup, RPS can provide information about the size with a simple setup. The main drawback of this kind of system is the lack of close contact between cells and electrodes when passing through the microchannel and the possible overlapping of two or more cells in the channel. One solution could be the use of narrower channels and sorting methods.

## 6. Conclusions

We have presented an overview of the status and challenges of the most relevant microfluidic systems for phytoplankton research. There is a clear need for such innovative microscale technology as several applications would benefit significantly. Lab on chip systems with microfluidic channels possess many advantages, such as fast measurements, high sensitivity, multifunctionality and possible portability. Despite this, these systems are mainly in the research stage, with few examples of commercial products. One reason could be the different modalities that can be used and their difficulty of integration. Each of these modalities offer unique capabilities but also pose some limitations. For this reason, sorting methods, multiparametric systems, multifunctional imaging and deep learning can bring solutions to actual issues. Nevertheless, some emerging proposals are opening new avenues towards enhanced phytoplankton measurement and the next decade seems promising.

## Figures and Tables

**Table 1:** Comparative table of different microfluidic technologies for phytoplankton research.

Modality	Source	Parameters Measured	Main Challenges and Limitations
Scattering	Visible light.	Cell size and shape.	Low intensity in comparison to fluorescence.
Raman scattering	>700 nm (avoid fluorescence).	Composition, nutrient contents.	Low intensity and long interrogation times.
Fluorescence	450 nm (clorophyl abs.), 550 nm (phycoerythrin abs.).	Concentrations of chlorophyll and other pigments, living status.	Highly variable due to their physiological conditions.
Imaging	Visible light.	Intracellular contents, size and surface roughness.	Limited throughput.
Time-stretch	Broadband fs-laser.	Same as imaging.	Complex/bulky setup.
Impedance	AC Voltage (1Hz-1MHz).	Cytoplasm conductivity and membrane capacitance.	Some species are difficult to model.
RPS	DC Voltage.	Size.	Lack of close contact and possible overlapping of cells.

## Data Availability

Data underlying the results presented in this paper are not publicly available at this time but may be obtained from the authors upon reasonable request.
